# Consensus in Gestational Diabetes MELLITUS: Looking for the Holy Grail

**DOI:** 10.3390/jcm7060123

**Published:** 2018-05-28

**Authors:** Mukesh M. Agarwal

**Affiliations:** Departments of Pathology and Medical Education, School of Medicine, California University of Science & Medicine, San Bernardino, CA 92408, USA; magarwal7@gmail.com; Tel.: +1-909-954-0032

**Keywords:** gestational diabetes mellitus, screening, diagnosis, consensus, controversy

## Abstract

The world’s pre-eminent diabetes, obstetric, endocrine, and health organizations advocate a plethora of diverse algorithms for the screening, diagnosis, management, and follow-up of gestational diabetes mellitus (GDM). Additionally, there are regional recommendations of local health societies. Several of these proposals for GDM are contentious because some of them were developed from unscientific studies, based on expert-opinion, catered to preserve resources, and subjectively modified for convenience. Due to the wide variety of choices available, the approach to GDM can be extremely diverse even within the same hospital. This lack of consensus creates major problems in addressing prevalence, complications, efficacy of treatment, and follow-up of GDM. Moreover, it becomes nearly impossible to compare the numerous studies. Furthermore, the lack of consensus confuses the health care providers of obstetric health who look to the experts for guidance. Therefore, a clear, objective, “evidence-based” global approach, which is simple, easy to follow, and validated by corroborative research, is crucial. We contend that, despite decades of research, a single acceptable global guideline is not yet on the horizon.

## 1. Introduction

*“So, oft in theologic wars*

*The disputants, I ween,*

*Rail on in utter ignorance*

*Of what each other mean,*

*And prate about an Elephant*

*Not one of them has seen”*

—John Godfrey Saxe

Gestational diabetes mellitus (GDM) is reminiscent of an old Indian fable about six blind men trying to describe an elephant after touching it. The blind man stroking the tail was certain the elephant was like a “rope”. Other blind men thought that the elephant was like a “pillar”, “fan”, “wall”, “snake”, and “solid pipe” depending on the part of the elephant that they felt. GDM, like the description of the elephant in the parable, remains without consensus [1]. Despite five decades of research, multiple international conferences and major collaborative trials most aspects of GDM remain controversial and chaotic [2,3].

Being multidisciplinary, a plethora of algorithms to screen, diagnose, treat, and follow-up patients with GDM are advocated by various international diabetes organizations, health societies, endocrine groups, and obstetric associations [4]. Often, there is a disagreement between a country’s obstetric and diabetes organization with each one preferring a different option for approaching GDM (e.g., American Diabetes Association, ADA [5], and American College of Gynecology and Obstetrics, ACOG [6]). Beside the international preeminent bodies, there are national professional health bodies in most countries (e.g., India, Brazil, Japan), which often develop GDM guidelines either based on local data or advocate what is feasible in their setting. Therefore, the approach to GDM screening and diagnosis has often remained parochial [7]. More than 17 GDM recommendations of professional bodies have recently been reviewed [8] even though many more are available. As a result, hospitals are often lost for choice about the way to approach GDM. For instance, the guidelines—all valid—that a hospital in the United Kingdom can follow, amongst others, are by the (a) National Institute for Health and Clinical Excellence, NICE [9], (b) Scottish Intercollegiate Guidelines network (SIGN) [10], (c) World Health Organization (WHO) [11], and (d) European Board and College of Obstetrics and Gynecology (EBCOG) [12].

The basis for using a guideline different from hospitals within the same geographic area is perplexing. Sometimes the reason for choosing any recommendation is often resource driven. A hospital may use a less demanding approach since it lacks the laboratory personnel, the hospital space, or has a shortage of funds. Often no potential reason is found. The logic of using an older guideline from an organization (e.g., WHO-1999 instead of WHO-2013) is unfathomable. Since this lack of harmony causes major problems in clinical practice, the need for consensus has been repeatedly expressed [13,14]. In this manuscript, we highlight some of the differences between various professional bodies. We analyze the reasons for these dissimilarities, summarize the attempts at consensus, and iterate the need for a global consensus.

## 2. Problems Caused by a Lack of Consensus

This heterogeneity in approaching GDM causes major problems, which are outlined below [15].

1. It is difficult to compare prevalence in different countries. In one study, due to variation in criteria and practices, the prevalence of GDM in 173 countries varied from <1–28% [16]: the different criteria used were a major contributor to the heterogeneity. Another study showed that, applying the diagnostic thresholds of eight major expert guidelines to the same glucose values of the diagnostic oral tolerance test (OGTT), the GDM prevalence varied from 9.2% to 45.3% depending on which criteria was used [17].
2. The GDM risk of progress to diabetes mellitus, type 2 (DM2) after delivery is difficult to estimate since different standards are used by various studies.
3. The variation in diverse ethnic groups is important since high-risk immigrants can be targeted. However, if the criteria for diagnosis vary, it is hard to estimate risk.
4. Published studies cannot be compared as the gold-standard for diagnoses vary. The 2008 effort by the United States Preventive Services Task Force (USPSTF) for screening of GDM acknowledged that the literature was limited by the lack of consistent standards for screening and diagnosis of GDM [18].
5. The association of perinatal and maternal complications in index pregnancy complicated GDM is difficult to determine objectively.
6. Since different criteria for diagnosing GDM exist, the effectiveness of different treatments cannot be compared.

## 3. Consensus on What Constitutes GDM

The origins of GDM are nebulous bordering on the apocryphal. Initially, there was much skepticism about the diagnosis of gestational diabetes. Some experts doubted its existence by calling it “a non-entity” [19] and “a diagnosis still looking for a disease” [20] even as late as the early 1990s. Therefore, earlier researchers wondered if GDM was a risk or a myth [21]. More recently, GDM has been touted as one of the diseases discovered due to medicalization [22] especially when using the newly formulated diagnostic criteria. In 1964, the original criteria for GDM diagnosis were based on the 100-g, 3-h OGTT. These criteria were established to predict DM2 after delivery. A decade and a half later, in 1979, the National Diabetes Data Group (NDDG) of the National Institute of Health, NIH accepted these criteria (after minor adjustments in diagnostic glucose thresholds). Additionally, they restricted the term GDM to glucose intolerance discovered (or developing) during pregnancy. Therefore, for many years, GDM was defined as hyperglycemia that was first discovered during pregnancy. However, due to the recent epidemic of DM2 spilling over to afflict numerous younger women in the child-bearing age, this traditional definition has been redefined. The WHO [11] classifies hyperglycemia in pregnancy as (a) diabetes mellitus in pregnancy and (b) GDM. The reasons for this segregation is that DM2 leads to more severe complications (e.g., congenital malformations) and needs to be treated aggressively and as early as possible. GDM signifies milder hyperglycemia occurring in the latter half of pregnancy, which frequently disappears after delivery in most patients. According to the ADA, GDM is diabetes diagnosed in the second or third trimester of pregnancy that is not Type 1 or Type 2 diabetes mellitus [5]. Therefore, over time, the definition of GDM has evolved but always plagued by a lack of consensus. Facetiously, GDM has been compared to the fairy tale, ‘The Emperor’s New Clothes’, where the emperor’s clothes (‘the guidelines’) were applauded by his courtiers (‘the preeminent organizations’) when there were really none (‘their dubious origin’) [23].

## 4. Consensus on Screening of GDM

Over the years, there have been exhaustive and heated debates about the screening for GDM. The questions that have been asked include: Is screening of pregnant women for GDM needed? Should we use only clinical risk factors for screening? Should we use the 50-g glucose challenge test (GCT) for screening? Should all women be screened (i.e., universal screening) with the OGTT directly? What is the most cost-effective way to screen for GDM? Should we use alternate screening methods like fasting plasma glucose and HBA1c? [24].

Screening for GDM does not meet the accepted criteria to screen for a disease, e.g., the United Kingdom (UK) National Screening Committee (NSC) guidelines [25]. However, all major health bodies such as the ADA [5] and the WHO [11] agree that screening for GDMis desirable. In 2002, an extensive analysis by the Health Technology Program [26] concluded, “On balance, the present evidence suggests that we should not have universal screening, but a highly selective policy based on age and weight of patients”. However, with more research being available, these ideas have been modified.

In 2008, the eminent United States Preventive Services Task Force (USPSTF), after a thorough review, determined that there was insufficient evidence to decide one way or another about the benefits and harms of screening for GDM. However, in 2014, after considering all the latest research, the USPSTF opined that asymptomatic women after 24 weeks of gestation should be screened for GDM. However, before 24 weeks, the evidence was insufficient [27].

Furthermore, there is debate about the best way to screen for GDM. Though originally screening via risk-factors (age, obesity, family history of DM, GDM in previous pregnancy, non-white race, previous miscarriages/stillbirths/fetal malformations, preeclampsia, and macrosomia) was widely recommended, many studies recommend the contrary [28]. Additionally, the clinical risk factors used for GDM vary in different studies. Therefore, studies are confounded by a double whammy: variation in risks used and differences in diagnostic criteria. The GCT is another popular screening test especially in North America. A recent editorial view (about the ideal way to screen for GDM) is that neither risk-factor nor GCT based screening is justified [29]. Their advice is to include the oral glucose tolerance test (OGTT) for use in both screening and diagnosis of GDM, which is recommended by The International Association of Diabetes and Pregnancy Study Groups (IADPSG). While the cost of screening increases and more women are labelled as GDM, the St. Carlos study shows that, in the long-term, it is cheaper due to fewer complications [30]. 

Many laboratory screening tests have been used for screening of GDM. They are direct glucose measurements (FPG, GCT) or indirect measurements of glucose (HBA1c, fructosamine). Newer markers (insulin, irisin, galanin, adiponectin, sex hormone-binding globulin, C-reactive protein, fibrinectin, glycosylated fibrinactin, ferritin, glycated CD59) especially in early pregnancy have been tried to predict GDM later in pregnancy. However, only GCT and FPG have shown some promise. The ideal method for screening of GDM has yet to be found or agreed upon.

## 5. Consensus on Diagnosis of GDM

More progress towards a consensus has been made in many countries. However, there is still a long way to go. Some of these diagnostic differences are shown in Table 1.

- Since the 1980s, both ACOG and the ADA recommendations were similar with some minor differences. However, their disparities were most evident in 2011 when the ADA endorsed universal screening with the 75-g OGTT (IADPSG criteria) while the ACOG did not. However, after the NIH sponsored conference [31], they accepted each other’s points of view, which is shown by their latest individual guidelines.
- In Europe, the WHO, which is another mover and shaker with global reach, decided in 1980 that it was easier to use the 75-g OGTT for both pregnant and non-pregnant women and adult males. This approach is convenient but inaccurate [32]. However, subsequently, multiple studies also showed that the subsequent WHO-1999 criteria for GDM diagnosis predicted complications in index pregnancy as well as DM2 after delivery. Due to its global acceptance, major local diabetes organizations across the world use 75-g OGGT as advocated by the WHO. However, despite great progress, there is still much heterogeneity in Europe, which is shown by a study from 28 European countries [33].
- In Australia, the Australasian Diabetes in Pregnancy Society (ADIPS) modified the WHO criteria and issued its own criteria (1991) based on expert opinion. These criteria were subsequently modified (1996). However, since 2013, the ADIPS issued new guidelines after considering the available evidence like HAPO accepted the WHO 2013 [11]. In New Zealand, guidelines of the ADIPS-1991 were accepted but the criteria for GDM diagnosis were made more restrictive. The reason was that less women would be diagnosed with GDM, which conserves New Zealand’s overextended funds. Even in 2018, despite all the newer data, the NZ guidelines have not been modified since 2014. Unlike ADIPS, they do not accept the IADPSG.
- The United Kingdom had three organizations with their own guidelines including NICE, SIGN, and Clinical Resource Efficiency Support Team (CREST). These organizations had a varying approach for the diagnosis and screening of GDM. NICE and SIGN have newer guidelines incorporating the new data. However, the CREST does not.
- The Canadian Diabetes Association (CDA) in their 2013 guidelines [34] suggest a modified approach to the IADPSG with minor differences. However, they still endorse the 50-g GCT, which the IADPSG does not. The Society of the Obstetricians and Gynecologists of Canada (SOGC) [35] differed from the CDA. However, in their 2016 guidelines, the SOGC has endorsed the CDA 2013 approach. Therefore, some of the disparities that are present between the obstetric and endocrine organizations in Canada over the last three decades do not exist anymore.
- Countries such as Japan, Brazil, and France followed their own recommendations or hybrid recommendations of the international organizations. However, many have accepted the IADPSG guidelines [36].

Therefore, it can be appreciated that most guidelines of the various professional committees are based primarily on consensus and expert opinion. However, after more research, more consensus has been achieved—an increasing trend over the last two decades.

## 6. Consensus on GDM Diagnosis in Early Pregnancy

Most guidelines recommend that GDM should be diagnosed between 24–28 weeks. However, the origin and the reason for choosing this time frame is not clear [37]. In the 1979 NDDG guidelines for GDM, no time frame was suggested. In 1996, the French College of Gynecologists and Obstetricians first suggested this time frame for low-risk women. In 1997, the Fourth International Workshop-Conference on GDM suggested testing in early pregnancy only on high-risk women and those with moderate risk between 24–28 weeks. In 2000, the ADA advised using Carpenter and Coustan criteria between 24–28 weeks [38]. Currently, there is a lack of consensus if GDM should be diagnosed in early pregnancy. In 2014, the USPSTF concluded that the evidence was not enough to assess the balance of benefits and harms of screening for GDM in asymptomatic pregnant women before 24 weeks of pregnancy [27]. However, higher first trimester FPG levels increase the risk of adverse pregnancy outcomes [39]. Therefore, the IADPSG recommended that FPG ≥ 5.1 mmol/L in early pregnancy should be considered as GDM. Some experts have cogently argued that GDM in pregnancy cannot be accepted since there are no controlled trials that address the benefits of diagnosing and treating GDM in early pregnancy [40]. Furthermore, high FPG in early pregnancy is poorly predictive of GDM in later months. Even the ADA does not support the diagnosis of GDM in early pregnancy. As will be appreciated, there is no consensus on GDM diagnosis in early pregnancy.

## 7. Consensus about OGTT as a Gold Standard for Diagnosis of GDM

The OGTT is a test with many problems. It is poorly reproducible [41], expensive, needs preparation, not physiologic, unpleasant, dependent on ethnicity, and given without consideration to body weight [42]. Additionally, it causes nausea and vomiting in pregnant women [43]. Ironically, there is consensus about using OGTT for GDM diagnosis. Every guideline agrees that the OGTT is the ‘gold standard’ for GDM diagnosis. In short, OGTT remains the cornerstone for diagnosis of GDM. However, the disagreement is about the glucose load used (75 g vs. 100 g), the number of samples collected (three vs. four), time needed for the test (two hours vs. three hours) and the thresholds used for diagnosis are the moot points with using the OGTT for diagnosing GDM. The IADPSG attempts to standardize the OGTT has met resistance despite much agreement among many countries and advisory organizations.

## 8. Consensus on Management of GDM

Management of GDM is crucial for preventing complications in the fetus and the mother. Once diagnosed, GDM is managed by diet alone when oral hypoglycemics and insulin do not work. It is unclear for how long non-pharmacologic treatment should be tried, the type of initial drugs (insulin vs. oral hypoglycemic medicine), dosage, and frequency of the initial therapy. The guidelines also vary regardling frequency of glucose monitoring, glycemic targets, and time of delivery [44].

## 9. Consensus on Follow-Up of GDM

GDM is a marker for future DM2 after delivery [45]. The incidence of postpartum diabetes varies widely from 15% to 70% according to the duration of follow-up, time of testing after delivery, ethnicity of the population, and diagnostic criteria for GDM [46]. Controversy surrounding the test used after delivery [47] and the screening frequency for follow-up post-partum. Generally, post-partum glucose testing is performed six weeks after delivery using either fasting glucose or a 75-g OGTT. Subsequently, follow-up testing is advised one to three years for normo-glycemic women. Clearly, a consensus will go a long way toward defining the post-natal prevalence of DM2 after GDM.

## 10. Efforts at Consensus

**GDM workshops:** In North America, five International Workshop-Conferences (1979–2005) on GDM tried to address multiple issues around GDM [48,49]. Due to these efforts, a degree of consensus was achieved [50] in North America.

- **HAPO trial:** Since the later part of the last decade, almost all the preeminent organizations and experts have been looking to the HAPO study [51]—a massive and expensive global effort—to be the panacea [52]. More than 23,000 pregnant women were enrolled in 15 field centers located in nine different countries between 2000–2006. However, even though it did resolve many issues, major differences and concerns still remain.
- **IADPSG criteria** were the collective wisdom of experts from 40 different countries to achieve consensus after the HAPO study. Therefore, the IADPSG criteria had the possibility to be accepted by the preeminent medical, endocrine, and health organizations worldwide. The IADPSG guideline is currently the most popular guideline for GDM. It has been accepted by many major world organizations like WHO, ADIPS, IDF, and FIGO [36]. CDA has not argued against it but would rather avoid them. However, the ACOG and the NIH consensus panel have reservations about the IADPSG recommendations [31].
- **NIH conference:** In 2013, the United States National Institutes of Health convened a Consensus Conference to consider all aspects of screening and diagnosis of gestational diabetes [31]. Again, the differences could not be resolved.
- **FIGO:** A major effort was undertaken by the International Federation of Gynecology and Obstetrics (FIGO), which has members from about 125 gynecology and obstetrics organizations worldwide to achieve consensus on GDM [53]. Their guidelines were widely endorsed by major international groups like EBCOG, the Society of Obstetricians and Gynecologists of Canada (SOGC), African Federation of Obstetrics and Gynecology (SAFOG), ADIPS, European Association of Perinatal Medicine, and Diabetes in Pregnancy Study Group of Latin America [54]. These guidelines acknowledge the difficulty in achieving a universal approach for GDM. This is because, among different countries, resources available are different, maternal and fetal complications during delivery vary in different locations, local findings are diverse, and the experts differ in using the resources. FIGO offers a practical solution by dividing the approach based on resources, i.e., countries with fully resourced settings, countries with fully resourced settings serving populations with high risk for GDM, and countries with medium resourced to low resourced settings. Therefore, every country can be on a trajectory toward improvement depending on its circumstances and yet be able to offer optimal care to its pregnant patients with hyperglycemia.

## 11. Conclusions

GDM continues to remain the proverbial riddle, wrapped in a mystery, inside an enigma [55]. However, despite all the tremendous efforts at consensus, the cup seems half empty rather than half full. The need for just one guideline has been iterated repeatedly by the experts who ironically continue to disagree. This tower of Babel mixed signal approach does not help the pregnant women who seek health care from the providers or the primary care givers who yearn for guidance from the experts. In many other areas of medicine, standardization has been achieved [56,57,58]. There is no reason why such harmony cannot be achieved with GDM. Now with decades of research and hindsight, we need that one clear, illuminating and unified global guideline for GDM:It is high time.

## Figures and Tables

**Table 1:** Comparison of some current diagnostic criteria of gestational diabetes.

Organization	Glucose Load, Grams	Glucose Thresholds (mmol/L)				Number of OGTT Values for Diagnosis ≥
		Fasting	1-h	2-h	3-h	
NDDG (ACOG)	100	5.8	10.5	9.2	8.0	2
C&C(ACOG)	100	5.3	10.0	8.6	7.8	2
IADPSG/WHO/ADIPS/FIGO/BSD	75	5.1	10.0	8.5	-	1
CDA	75	5.3	10.6	9.0	-	1
NICE	75	5.6	-	7.8	-	1
DIPSI	75	-	-	7.8		-

ACOG: American College of Obstetrics and Gynecology; ADA: American Diabetes Organization; ADIPS: Australian Diabetes in Pregnancy Society; BSD: Brazilian Society of Diabetes; CDA: Canadian Diabetes Association; C&C: Carpenter and Coustan; DIPSI: Diabetes in Pregnancy Study group in India; FIGO: The International Federation of Gynecology and Obstetrics; FPG: Fasting plasma glucose; IADPSG: The International Association of Diabetes and Pregnancy Study Groups; NDDG: National Diabetes Data Group; NICE: National Institute for Health and Care Excellence; WHO: World Health Organization.
